# Whole Cell Cross-Linking to Discover Host–Microbe Protein Cognate Receptor/Ligand Pairs

**DOI:** 10.3389/fmicb.2018.01585

**Published:** 2018-07-19

**Authors:** Bart C. Weimer, Poyin Chen, Prerak T. Desai, Dong Chen, Jigna Shah

**Affiliations:** ^1^Department of Population Health and Reproduction, School of Veterinary Medicine, University of California, Davis, Davis, CA, United States; ^2^Department of Dietetics, Nutrition and Food Sciences, Utah State University, Logan, UT, United States; ^3^Department of Biology, Utah State University, Logan, UT, United States

**Keywords:** whole cell cross linking, *Salmonella*, receptor/ligand, fibronectin, SLAP domain

## Abstract

Bacterial surface ligands mediate interactions with the host cell during association that determines the specific outcome for the host–microbe association. The association begins with receptors on the host cell binding ligands on the microbial cell to form a partnership that initiates responses in both cells. Methods to determine the specific cognate partnerships are lacking. Determining these molecular interactions between the host and microbial surfaces are difficult, yet crucial in defining biologically important events that are triggered during association of the microbiome, and critical in defining the initiating signal from the host membrane that results in pathology or commensal association. In this study, we designed an approach to discover cognate host–microbe receptor/ligand pairs using a covalent cross-linking strategy with whole cells. Protein/protein cross-linking occurred when the interacting molecules were within 9–12 Å, allowing for identification of specific pairs of proteins from the host and microbe that define the molecular interaction during association. To validate the method three different bacteria with three previously known protein/protein partnerships were examined. The exact interactions were confirmed and led to discovery of additional partnerships that were not recognized as cognate partners, but were previously reported to be involved in bacterial interactions. Additionally, three unknown receptor/ligand partners were discovered and validated with *in vitro* infection assays by blocking the putative host receptor and deleting the bacterial ligand. Subsequently, *Salmonella enterica sv.* Typhimurium was cross-linked to differentiated colonic epithelial cells (caco-2) to discover four previously unknown host receptors bound to three previously undefined host ligands for *Salmonella*. This approach resulted in *a priori* discovery of previously unknown and biologically important molecules for host/microbe association that were casually reported to mediate bacterial invasion. The whole cell cross-linking approach promises to enable discovery of possible targets to modulate interaction of the microbiome with the host that are important in infection and commensalism, both of with initiate a host response.

## Introduction

Bacterial association with mammals is a complex co-evolutionary partnership that has evolved over billions of years (McFall-Ngai et al., 2013). Co-selection of the microbiome and the community membership, diversity, and metabolic capability has a profound impact on the health status of the host. Selective pressures over time that result in the emergence of host receptor and bacterial ligand partnerships that have functionally co-evolved (Kline et al., 2009). While specific host receptors are defined for specific pathogenic microbes, many more specific partnerships remain to be discovered to fully explain host/microbe association, infection mechanisms, and microbiome commensalism (Kingsley et al., 2002; Tukel et al., 2005; Kisiela et al., 2006). Host–microbe receptor/ligand partnerships are one of the most critical determinants that control bacterial host range (Lindstedt et al., 1991), have a role in bacterial tissue tropism (Fitzhenry et al., 2002), and are the initiating step in pathogenesis (Arabyan et al., 2016, 2017a,b; Park et al., 2016).

Bacterial adhesion is the first step of the bacterial association process and initiates signal transduction routes in the host in response to the microbial ligands and the exact host receptor with many possible partnerships between the host and the microbe (Ribet and Cossart, 2015). In the case of bacteria, such as *Listeria monocytogenes*, *Lactobacillus*, and *Salmonella enterica* (Cossart and Sansonetti, 2004; Arabyan et al., 2016; Park et al., 2016), glycan association, digestion, subsequent access to membrane-embedded receptors are the prelude to host cell invasion that causes gastrointestinal infection via bacterial ligand binding, tissue invasion, possible systemic disease, secondary infections, and in some cases long-term carrier states that are emerging as an underlying cause of chronic inflammatory diseases (Tlaskalova-Hogenova et al., 2004; Ribet and Cossart, 2015). Likewise, recognition of host-cell surface molecules by commensal and probiotic bacteria is also important for the observed health benefits of bacterial association with humans (Lebeer et al., 2010). Probiotic bacteria initiate a pro-inflammatory or an anti-inflammatory response in the host based on the type of microbial ligands bound by host receptors (Swamy et al., 2010). Consequently, identification and characterization of receptor/ligand pairs is an important area of study that is poised to provide new discoveries and expand understanding of how bacterial association modulates mechanisms associated with pathogenesis, microbiome membership and metabolism, and govern the overall effect of the microbiome on health and disease in humans and animals.

Control of bacterial infection is becoming increasingly challenging with rising antimicrobial resistance (Gilbert et al., 2007; Hu et al., 2016), multi-drug resistance from wildlife sources (Weis et al., 2016, 2017), and emergence of hypervirulent strains via livestock (Heithoff et al., 2012). Rapid emergence of antibacterial resistance urgently renews the call for alternative compounds and strategies to control infectious disease agents that has yet to be met. An alternate strategy is disruption of pathogen adhesion is a strategy that will slow or stop disease progression via microbiome association blocking. Such strategies based on glycosylated molecules, such as lactoferrin (Barboza et al., 2012), lysozyme (Maga et al., 2012, 2013), or prebiotic oligosaccharides (Marcobal et al., 2011; Ng et al., 2013; Ferreyra et al., 2014) demonstrate that specific molecules mediate microbiome membership and cooperation between microbes and metabolites to change the host response. However, to fully harness the potential of microbiome association determinants, discovery and characterization of receptor/ligand partners are needed. Additionally, microbial adhesion molecules that consistently pair with host receptors indicating that these protein portions are exposed to other cells and ready to be presented to the immune system are potential vaccine candidates. Langermann et al. (1997) successfully demonstrated this approach using an adhesin-based vaccine to reduce *in vivo* colonization of *Escherichia coli* by >99% in a murine model. Identifying the cognate receptor/ligand partnerships used by pathogenic bacteria to bind and invade host cells is the first step in development of therapies that will reduce pathogen infection, acute systemic disease, bacterial shedding, and potentially bacterial-associated chronic disease (e.g., chronic inflammation). Consequently, identification and characterization of receptor/ligand pairs is an important area of study that is poised to provide new discoveries and expand understanding of how bacterial association modulates mechanisms associated with pathogenesis.

Identification of receptor/ligand partnerships is a difficult task because reproduction of the disease-specific conditions is needed to avoid false positive associations. No methods exist that specifically define the respective molecules in the partnership during whole cell adhesion or active infections. Even though reports of bacterial virulence molecules are common, rarely is the cognate host receptor/ligand partner determined. One approach to define the partnership is to screen libraries of tagged mutants for defects in adhesion/colonization phenotype (Maroncle et al., 2002). Unfortunately, this approach does not reveal the identity of the cognate host receptor. While affinity pull down approaches expand the listing of partnerships, identity of at least one host binding partners must be known before using this approach (Cabanes et al., 2005) and is prone to false positives. Considering the high redundancy of bacterial ligand molecules on the surface of bacteria, screening mutant libraries where a single locus is disrupted lacks the ability to determine the complex multi-factorial interactions between the host and the microbe cell surfaces, which leads to an excruciatingly slow discovery of receptor/ligand partnerships, that inhibits progress to find new targets to reduce bacterial pathogen association. Conversely, in the case of probiotic bacteria it is useful to increase the association and may increase the competitive advantage of these organisms via specific molecules that provide single protein (i.e., gene) effects (Johnson et al., 2015). This study describes a novel method to discover cognate receptor/ligand partnerships used by pathogen and probiotic bacteria to bind the host epithelial cells during active association and invasion.

The approach used in study relied on a NHS-ester moiety to preferentially and covalently bind two proteins within 9–12 Å of one another (Hermanson, 1996). To initiate cross-linking process one protein or cell was treated with sulfo-SBED leaving two other functional groups exposed for use later in cross-linking and isolation of the protein from the partnership for identification and verification. Once a cell is labeled with the reagent via a specific protein another cell is added, incubated, and the ligand is cross-linked using photo-activation of the aryl azide moiety to bind interacting molecules within 9–12 Å. This process allows for protein–protein interactions and subsequent covalent cross-linking under physiological conditions between two protein molecules that are intimately interacting on the cell surface. The cross-linking strategy used here included a disulfide linkage that transfers the biotin moiety from the molecule initially labeled to its interacting partner when chemically reduced to ensure the interacting proteins are partners in the host-to-bacterium binding. All these properties were exploited for verification of intimate and specific protein/protein interaction needed during bacterial adhesion. We demonstrated the capability of this approach by purifying proteins from intact, viable cells in combination with pathogenic and probiotic bacteria during biologically relevant interaction conditions with human colonic epithelial (Caco-2) cells. Expanding the catalog of known host–microbe receptor/ligand pairs contributes to advancement of host–microbe interaction research as well as the development of potential therapies toward treating bacterial diseases.

## Materials and Methods

### Cell Culture and Bacterial Strains

Caco-2 cells were obtained from ATCC (HTB-37, Manassas, VA, United States) and cultured as recommended by ATCC and grown as previously described (He et al., 2013; Shah et al., 2014) with passage numbers 22–30. In brief, cells were plated at a density of 10^5^/cm^2^ in either a T25 or a 96-well plate after differentiation (Ouwehand and Salminen, 2003). Cells were maintained in DMEM/high modified (Thermo Scientific, Rockford, IL, United States) with 16.6% fetal bovine serum (FBS) (HyClone Laboratories, Logan, UT, United States), non-essential amino acids (Thermo Scientific, Rockford, IL, United States), 10 mM MOPS (Sigma-Aldrich Corp., St. Louis, MO, United States), 10 mM TES (Sigma-Aldrich Corp., St. Louis, MO, United States), 15 mM HEPES (Sigma-Aldrich Corp., St. Louis, MO, United States), and 2 mM NaH_2_PO_4_ (Sigma-Aldrich Corp., St. Louis, MO, United States). Cells were considered to be differentiated 14 days post confluence (Ouwehand and Salminen, 2003), and used for the adhesion and cross-linking assays. *Lactobacillus acidophilus* cultures were obtained from ATCC (700396, Manassas, VA, United States) and grown microaerophilically on MRS media at 37°C as described by Chou and Weimer (1999 #3704). *Salmonella* Typhimurium and *Escherichia coli* were obtained from ATCC (14028, 43895, Manassas, VA, United States). *Salmonella* and *E. coli* were grown aerobically on LB media at 37°C.

### Labeling Bacterial Cells and Purified Proteins With Sulfo-SBED

The bacterial surface was labeled with Sulfo-*N*-hydroxysuccinimidyl-2-(6-[biotinamido]-2-(*p*-azido benzamido)-hexanoamido) ethy-1,3′-dithioproprionate (Sulfo-SBED) (Thermo Fisher Scientific, Rockford, IL, United States). The total protein content on ∼10^9^ bacteria’s surface was measured using the BCA protein assay (Thermo Fisher Scientific, Rockford, IL, United States). Assuming an average molecular weight of 60,000 Da for proteins, a 10-fold molar excess Sulfo-SBED (dissolved in DMSO at 50 μg/μl) of the determined protein concentration, was added to 10^9^ bacteria/ml suspended in 1 ml of Tyrode’s buffer (140 mM NaCl, 5 mM KCl, 1 mM CaCl_2_, 1 mM MgCl_2_, 10 mM glucose, 10 mM sodium pyruvate, 10 mM HEPES, pH 7.4) The labeling reaction was conducted in dark on ice for 45 min with intermittent shaking. After incubation, the reaction was quenched by adding twofold molar excess glycine compared to Sulfo-SBED. The bacteria were washed twice with Tyrodes buffer by centrifugation at 6000 × *g* for 2 min and resuspended in 1 ml Tyrodes buffer.

Fibronectin (Sigma-Aldrich Corp., St. Louis, MO, United States), fibrinogen (Sigma-Aldrich Corp., St. Louis, MO, United States), and amyloid precursor protein (APP; Novus Biologicals, Littleton, CO, United States) were dissolved in Tyrodes buffer at 1 mg/ml and were labeled with 10-fold molar excess of sulfo-SBED as described above. After quenching the labeling reaction, the proteins were desalted with Tyrodes buffer using Microcon YM-30 (Millipore, Billerica, MA, United States) as described by the manufacturer’s instructions and resuspended at a concentration of ∼1 mg/ml.

### Protein Identification

The cell lysate (50 μl) was diluted with 50 μl of 2× Laemmli buffer (Shapiro et al., 1967) (52.5 mM Tri–HCL, pH 6.8, 25% glycerol, 2% SDS, 0.02% bromophenol blue) with or without the addition of 150 mM DTT and heated at 95°C for 10 min. Subsequently, the lysate was centrifuged at 12,000 × g in a micro-centrifuge for 5 min and the proteins (50 μl) in the supernatant were separated by SDS–PAGE using the Mini-PROTEAN electrophoresis system (Bio-Rad Laboratories, Hercules, CA, United States) as described by manufacturer’s insert at a constant current of 30 mAmp per gel, using 4–20% precast Tris–HCL Gels (Bio-Rad Laboratories, Hercules, CA, United States). The gels were stained overnight with Imperial protein stain (Thermo Fisher Scientific, Rockford, IL, United States) as per the manufacturer’s protocol. The gels resolved in reducing and non-reducing conditions were imaged using the Kodak Image Station 2000R (Carestream Health). The images for gels were visually compared for missing bands in the reduced gels that were targeted for excision and protein identification as using LC–MS/MS at the Center for Integrated BioSystems, Utah State University (Logan, UT, United States) (Liang et al., 2006; **Supplementary Figure S1**).

### *Salmonella* Gene Deletion

Deletion mutants in *Salmonella* were constructed as described by Datsenko and Wanner (2000). Briefly, mini-prep kit (Qiagen, Valencia, CA, United States) was used to isolate plasmid pKD46 containing ampicillin resistance and λ Red recombinase genes from *E. coli* BW25141 (CGSC 7634), plasmid pKD3 containing chloramphenicol resistance gene from *E. coli* BW25141 (CGSC 7631), and plasmid pKD4 containing kanamycin resistance gene from *E. coli* BW25141 (CGSC 7632). This plasmid pKD46 was electroporated in to *S.* Typhimurium and transformants were selected by growth on LB agar containing 100 μg/ml ampicillin (Sigma-Aldrich Corp., St. Louis, MO, United States). *S.* Typhimurium containing pKD46 was grown in LB broth in the presence of 100 μg/ml ampicillin and 100 mM L-arabinose to induce λ Red recombinase production. The chloramphenicol or kanamycin resistance genes were amplified using plasmid pKD3 or pKD4 templates, respectively, to ensure exact deletion sites. The primers used for amplification of the gene encoding the phage tail-like protein (STM2699) were STM2699 P1 and STM2699 P2. The primers used for amplification of the gene encoding for the integration host factor protein (*ihfA*, STM14_1626) were STM14_1626 P1 and STM14_1626 P2. The purified PCR products were electroporated into *S.* Typhimurium with induced λ Red recombinase. The transformants were selected on LB agar either with 10 μg/ml chloramphenicol or 40 μg/ml kanamycin. The gene deletion for ΔSTM2699 was confirmed by PCR using primers STM2699 J1, STM2699 J2, STM2699 F, and STM2699 R. The gene deletion for ΔSTM14_1626 was confirmed by PCR using primers STM14_1626 J1, STM14_1626 J2, STM14_1626 F, and STM14_1626 R. All primer sequences are available in **Supplementary Table S1**.

### Verification of Microbial Ligands With Purified Proteins

Microbial cells (∼10^9^ cfu/ml) were incubated with 1 ml of Sulfo-SBED labeled fibronectin, fibrinogen, or APP (1 mg/ml) for 30 min at 37°C. Subsequently, the suspension was placed under a 15-watt UV lamp (302 nm) at a distance of 5 cm for 10 min for cross-linking. The cells were washed twice with 1 ml Tyrodes buffer by centrifugation at 6000 × *g* for 2 min and resuspended in 500 μl lysis buffer (0.1% Triton, 150 mM DTT) and 250 μl of glass beads (0.1 mm) (BioSpec Products, Inc., Bartlesville, OK, United States). The samples were homogenized in a Mini-Beadbeater (BioSpec Products, Inc., Bartlesville, OK, United States) by giving three pulses at full speed for 30 s with intermittent 1 min incubation on ice (Chou and Weimer, 1999; Chou et al., 2001). The free biotin on the Sulfo-SBED reagent from the reduced non-cross-linked proteins was removed by passing the lysate through an YM3 Microcon ultrafiltration module (Millipore, Billerica, MA, United States) as described in manufacturer’s instructions. The retantate (volume brought up to 800 μl with Tyrodes buffer) (De Ridder et al., 1975) from the Microcon module was incubated with ∼30 avidin-coated glass beads (3 mm; Xenopore Corp., Hawthorne, NJ, United States) for 30 min on a shaking platform to capture the biotinylated proteins. The glass beads were subsequently washed three times with 5 ml of wash buffer (50 mM Tris, 1.5 M NaCl, pH adjusted to 7.2) to remove non-specifically bound proteins. The proteins on the washed beads were digested overnight using 300 ng of proteomics grade trypsin (Sigma-Aldrich Corp., St. Louis, MO, United States) in 1 ml of 100 mM ammonium bicarbonate buffer (Liang et al., 2006). The digested proteins were concentrated using a speedvac (Thermo Fisher Scientific, Rockford, IL, United States) to final volume of 50 μl and submitted to the Center for Integrated BioSystems, Utah State University (Logan, UT, United States) (Liang et al., 2006; Boudina et al., 2009; Wende et al., 2015) or the Mass Spectrometry and Proteomics Core Facility, University of Utah (Salt Lake City, UT, United States) for protein identification by LC/MS/MS. Analysis of *Salmonella* ligands to APP set the peptide mass tolerance at ±6 ppm and fragment mass tolerance at ±0.6 Da via LC/MS/MS. Peptides were identified using a MASCOT with the NCBInr database and confirmed against a custom *Salmonella* protein database constructed from the exact bacterial genome used in this experiment (NC_016856). The entire labeling protocol was repeated using unlabeled host binding components as negative controls in three biological replicates.

### Identification of Host Receptors by Whole Cell Cross-Linking

Prior to whole cell cross-linking the cells were maintained in osmotically balanced buffer to maintain cell membrane integrity and subsequently reduce interference from intracellular proteins during the whole cell experiment. Sulfo-SBED labeled bacteria (10^9^ bacteria/ml) were interacted with ∼10^6^ Caco-2 cells grown in a T25 in a final volume of 3.5 ml Tyrodes buffer for 60 min at 37°C. At the end of 60 min incubation, the bacterial suspension was aspirated from the flask and the host cell flask was placed under a 15-watt UV lamp (302 nm) at a distance of 5 cm for 10 min. The cross-linked Caco-2 cells and associated bacteria were resuspended in 500 μl of lysis buffer [8 M urea, 6.0% ampholytes pH range (3–10) (Bio-Rad Laboratories, Hercules, CA, United States), 0.4% CHAPS, 0.25% Triton 100, 0.15% *n*-dodecyl-B-β-D-maltoside, 0.002% bromophenol blue] and 250 μl of glass beads (0.1 mm, BioSpec Products, Inc., Bartlesville, OK, United States). The samples were homogenized in a Mini-Beadbeater (BioSpec Products, Inc., Bartlesville, OK, United States) by giving three pulses at full speed for 30 s with intermittent 1 min incubation on ice. The samples were stored at -70°C until further use. Cross-linked extracted samples were processed with 2D gels and LC/MS/MS for protein identification. InterProScan (Markowitz et al., 2006) was used to identify each protein in the partnership to verify the accuracy of the identified pairs.

### Cross-Linked Protein Selection Using 2D Gel

The cross-linked samples were thawed and centrifuged at 12,000 X g in a microcentrifuge and the supernatant was used for 2D gel analyses (Liang et al., 2006; Pate et al., 2007, 2008). Isoelectric focusing (IEF) was done using 50 μl of sample in tube gels using the Model 175 tube cell (Bio-Rad Laboratories, Hercules, CA, United States) as per the manufacturer’s instructions. In brief, 4% polyacrylamide gels were cast in 1 mm diameter tubes to a height of 11 cm. Samples (50 μl) were loaded in to the tubes and electrophoresed at 200 V for 2 h, 400 V for 4 h, and finally at 800 V for 8 h. After the IEF run, gels were extruded from the tubes and equilibrated in transfer buffer (3% SDS, 0.07 M Tris–HCL) with or without 150 mM DTT for 15 min. The gels equilibrated in presence of DTT were subsequently alkylated in transfer buffer using 150 mM iodoacetamide for 15 min. The tube gels were then resolved in the second dimension using the Criterion electrophoresis system (Bio-Rad Laboratories, Hercules, CA, United States) exactly as per manufacturer’s recommendation at a constant current of 45 mAmp per gel using 4–20% precast Tris–HCL Gels (#345-0104, Bio-Rad Laboratories, Hercules, CA, United States). The gels were stained overnight with Imperial protein stain (Thermo Fisher Scientific, Rockford, IL, United States) exactly as per manufacturer’s recommendations.

The gels resolved under reducing and non-reducing conditions were imaged using the Kodak Image Station 2000R (Carestream Health, Rochester, NY, United States). The images were compared and spots missing in the reducing gels but present in the non-reduced gel were identified. Protein spots were selected if: (1) the spot disappeared in reduced conditions; (2) had host and microbe protein in the same spot; (3) the sum of molecular weight of the identified proteins matched the observed molecular weight on the gel. The gel spots were picked, in-gel digested, and the proteins identified using LC–MS/MS at the Center for Integrated BioSystems, Utah State University (Logan, UT, United States; **Supplementary Figure S1**).

### Bioinformatic Analysis of LBA0222

To determine if the additional proteins found to bind fibronectin were relevant and indicative of a robust method, an investigation of the characteristics for LBA0222 was done using displayed Dendroscope (SSDB paralog search, SSDB domain analysis) (Aoki and Kanehisa, 2005; Jensen et al., 2009; Huson and Scornavacca, 2012) to discover the domain conservation.

### Determination of Total Host Associated Bacteria

The role of specific receptors and ligands in adhesion and invasion of the host by *Salmonella* was assayed by determining the changes in the amount of total host (Caco-2 cells) associated *Salmonella* after to receptor blocking by specific antibodies to specific host proteins identified by cross-linking. Caco-2 cells were cultured as described above, except it was done in a 96-well plate format. The bacteria were used after two transfers for the adhesion assays (Chen et al., 2017). Bacterial cells were collected from 2 ml of media after growth for 14 h, washed twice with an equal volume of PBS, and re-suspended at ∼10^8^ cfu/ml, in DMEM/high modified with 1× non-essential amino acids, 10 mM MOPS, 10 mM TES, 15 mM HEPES, and 2 mM NaH_2_PO_4_ but without the FBS. Caco-2 cells were incubated with dilutions of anti-SPTAN Ab (1:1000, 1:2000, 1:4000, and 1:8000) (Novus Biologicals, Littleton, CO, United States). Anti-APP Ab was used to block APP at a final dilution of 1:800, after an optimization that used additional concentrations (1:200, 1:400, 1:800, 1:1600), with an incubation of 60 min in a final volume of 50 μl at 37°C in 5% CO_2_. At the end of 60 min, the Caco-2 cells were infected with *Salmonella* as previously described (Arabyan et al., 2016) (MOI 1:100) and incubated for 60 min at 37°C in 5% CO_2_. The bacterial cell suspension was aspirated and the Caco-2 monolayer was washed thrice with 200 μl of Tyrodes to remove non-adhered bacterial cells from the monolayer. Intracellular bacteria were further quantified by a 2 h incubation in 40 μg/ml gentamicin solution (Sigma-Aldrich, St. Louis, MO, United States) and washed thrice with 200 μl of Tyrodes buffer to remove dead bacterial cells from the surface of the monolayer (Elsinghorst, 1994; He et al., 2013; Shah et al., 2014; Arabyan et al., 2016). DNA extraction buffer (AEX Chemunex, France; 50 μl) was used to lyse the monolayer and the bacteria associated with the host, and incubated at 37°C for 15 min followed by 95°C for 15 min. The resulting cell lysate was used to determine the number of bacteria associated with the Caco-2 cells. Quantitative analysis was done using qPCR with a CFX 96 Real Time System (Bio-Rad, Hercules, CA, United States). Reactions were performed in a final volume of 25 μl including 1 μl of cell lysate, 100 nM of PCR primers, and iQ SYBR Green Supermix (Bio-Rad, Hercules, CA, United States) as per manufacturer’s instructions. The primers used for the amplification are listed in **Supplementary Table S1**. The reaction parameters consisted of denaturation step at 95°C for 5 min, followed by 40 cycles of denaturation, annealing, and extension at 95°C for 15 s, 56°C for 30 s, 72°C for 30 s, respectively, and a final extension at 72°C for 1 min. The product was verified using a melt curve analysis from 50 to 95°C with a transition rate of 0.2°C/s. The number of bacterial cells and Caco-2 cells present in each well were determined by using a standard curve of C_T_ vs. Log_10_ cfu and concentration of bacteria per Caco-2 was calculated. The data were normalized relative to control wells that were not given Ab treatment. The experiment was done in four biological replicates. Effects of the treatments were assessed using one-way ANOVA, and individual means were compared to that of control by Dunnett’s multiple comparison test. Means were considered significantly different at *p* < 0.05.

### APP Degradation by *Salmonella*

Early stationary phase (14 h) *Salmonella* cultures and the corresponding spent supernatant were interacted with purified APP protein (NBP1-99026, Novus Biologicals, Littleton, CO, United States) for 30 min at 37°C. Cells were suspended in 100 mM Tris–HCl buffer (pH 7.2) while spent Luria Broth (LB broth; BD Difco, Franklin Lakes, NJ, United States) was used as the spent supernatant. Following incubation, APP suspensions were examined using a 4–12% gradient SDS-page gel and stained with SYPRO Ruby Protein Gel Stain (Thermo Fisher Scientific, Petaluma, CA, United States).

## Results

### Verification of Fibronectin Binding Proteins Using *Lactobacillus acidophilus* NCFM

Actively growing *L. acidophilus* NCFM was incubated with Sulfo-SBED-labeled fibronectin beads and cross-linked. LC–MS/MS analysis revealed two proteins from the same operon – cell separation protein (CdpA) and an uncharacterized protein encoded by LBA0222 – were bound to fibronectin. Additionally, four ribosomal proteins were cross-linked to fibronectin (**Table 1**). A negative control in which unlabeled fibronectin was interacted with the bacterial cells identified no proteins. This verified previous observations confirming CdpA (Johnson et al., 2015) and found additional bacterial proteins.

**Table 1:** Fibronectin binding proteins identified in *L. acidophilus* NCFM using whole cell cross-linking with an individual protein.

Locus ID	Protein	Unique peptides	MASCOT score	Comment
LBA0222	Hypothetical protein	3	141	See **Supplementary Data**
LBA0223	Cell separation protein, CdpA	2	141	Altermann et al. (2004) reported Caco-2 binding. This protein contains an ECM binding domain, a transmembrane domain, and a signal peptide
LBA0291	50S ribosomal protein L3	2	137	Found on the bacterial cell surface (Schwedler-Breitenreuter et al., 1985; Severin et al., 2007)
LBA0315	30S ribosomal protein S13	2	146	Found on the bacterial cell surface (Schwedler-Breitenreuter et al., 1985; Severin et al., 2007)
LBA0324	30S ribosomal protein S9	3	126	Found on the bacterial cell surface (Schwedler-Breitenreuter et al., 1985; Severin et al., 2007)
LBA0786	30S ribosomal protein S4	2	104	Found on the bacterial cell surface (Schwedler-Breitenreuter et al., 1985; Severin et al., 2007)


*A negative control using fibronectin lacking the Sulfo-SBED interacted with *L. acidophilus* NCFM cells yielded no proteins for identification.*

Functional analysis of CdpA revealed that this protein accounts for ∼80% of the adhesion capability of NCFM to gut epithelial cells (Altermann et al., 2004). Predicted homologs of this protein were found in *Lactobacillus* spp. containing orthologs of CdpA, S-layer proteins, and levansucrase (**Supplementary Figure S2**), confirming this approach found specific proteins that mediate adhesion of lactobacilli.

The hypothetical protein found during cross-linking is in an operon with *cdpA* that is unique to NCFM. Protein network analysis of CdpA found two predicted functional partners: two bacterial surface layer proteins – LBA0220 and LBA0221 that are in a single operon – as well as ribose-*p*-pyrophosphokinase (Prs) (**Supplementary Figure S3**), again confirming published results. Protein domain analysis (Cserzo et al., 1997; Aoki and Kanehisa, 2005; Jensen et al., 2009) found LBA0222 and CdpA to contain SLAP and FIVAR protein domains (**Supplementary Figure S4**-48pc). SLAP domains are found in numerous bacterial cell surface proteins, including S-layer proteins, amidases, and cell separation proteins, suggesting this domain is important in host receptor binding. As they are often found in combination with glycosyl–hydrolase domains, further suggesting that they are involved in hydrolyzing the host glycan as recently found to be important in *Salmonella* for invasion (Arabyan et al., 2016).

The protein encoded by the LBA0222 locus was also cross-linked to fibrinogen, and is in the same operon as *cdpA* (**Supplementary Figure S4**). Bioinformatic analysis of the LBA0222 protein determined that homologs were found only in other lactobacilli and one genome of *Chlamydia* (**Supplementary Figure S2**). The genetic neighborhood was unique to NCFM and is in the same operon as CdpA, as well as other organisms (**Supplementary Figure S3**). These observations found the exact results from previous reports (Johnson et al., 2013, 2015) in NCFM. This recapitulation of the results led to expansion of the approach to pathogenic bacteria with pure proteins suspected of binding pathogenic enteric bacteria where host interaction is more complex and less well described.

### Identification of Bacterial Ligands in Enteric Pathogens

As with lactobacilli, we initiated verification of the methods using purified proteins. Each 2D gel spot contained fibrinogen as well as a bacterial protein (**Table 2** and **Supplementary Figure S1**). Detection of *S*. Typhimurium and *E. coli* ligands to fibrinogen revealed one candidate spot on 2D gels for each organism. The cross-linked protein complex from *S.* Typhimurium LT2 contained fibrinogen and murein lipoprotein (*lpp*) [STM1376 (*lppB*), STM1377 (*lpp*)], while the cross-linked 2D gel spot from *E. coli* contained fibrinogen and a universal stress protein B (*uspB*; b3494). This confirmed previously known ligands associated with pathogenesis for these organisms and was shown that deletion of *lpp* resulted in lower infectivity (Sha et al., 2004; Daley et al., 2005).

**Table 2:** Fibrinogen-binding protein identified in *E. coli* and *S. sv.* Typhimurium after cross-linking.

Bacterium	Locus ID	Organism	Protein annotation	Unique peptides	Mw (Da)	PLGS score
*E. coli*	B3494	*E. coli* K12	Universal stress protein B	2	13,018	103
	FGA	*H. sapiens*	Fibrinogen alpha chain	22	69,713	424
	FGB	*H. sapiens*	Fibrinogen beta chain	22	55,892	320
	FGG	*H. sapiens*	Fibrinogen gamma chain	20	49,464	170
S. sv. Typhimurium	STM1377	*S. sv.* Typhimurium	Murein lipoprotein (Lpp)	3	8,386	320
	FGA	*H. sapiens*	Fibrinogen alpha chain	27	69,713	2408
	FGB	*H. sapiens*	Fibrinogen beta chain	18	55,892	1596
	FGG	*H. sapiens*	Fibrinogen gamma chain	15	49,464	1239


*PLGS score is calculated by the Protein Lynx Global Server (PLGS 2.2.5) software using a Monte Carlo algorithm and is a statistical measure of accuracy of assignation. A higher score indicates greater confidence in protein identity (Wright et al., 2009). The proteins were identified from a band in the gel which had an apparent molecular weight of >200 kDa.*

Secondarily, *S.* Typhimurium was cross-linked to purified APP, which is a membrane-associated host protein whose proteolytic product recently emerged as a possible protective factor to enteric pathogens, including *Salmonella* (Kumar et al., 2016). Cross-linking resulted in identification of 39 bacterial proteins to be associated with APP. Of those identified, 15 had a sequence query match of sufficient strength (i.e., >50) to be considered viable for investigation (**Table 3**). Those ligands included bacterial membrane-associated proteins, entericidin B (EcnB; STM4336), FtsH protease regulator (HflK; STM4363), and integration host factor (IhfA; STM1339). Homologs of these proteins have been characterized in *E. coli* but not in relation to host interaction and do not have host association receptor proteins reported (Bishop et al., 1998 #2586; Kihara et al., 1998 #2588; Bandyopadhyay et al., 2010 #2589). Use of purified proteins with these pathogens verified bacterial proteins known to facilitate bacterial association using the approach using two different purified host proteins. Consequently, we proceeded to use whole bacterial and host cells in cross-linking experiments.

**Table 3:** Binding proteins identified in *S. sv.* Typhimurium after cross-linking with App bound to a glass bead.

Protein identification	Locus ID	Queries matched	% Sequence coverage	Score
Entericidin B	*ecnB*	1	43.75 %	115
30S ribosomal protein S2	*rpsB*	3	15.76 %	93
Putative cytoplasmic protein YciF	*yciF*	1	7.75 %	78
FtsH protease regulator HflK	*hflK*	2	6.21 %	73
Inner membrane protein	*yqjD*	2	30.69 %	67
50S ribosomal protein L10	*rplJ*	2	16.36 %	65
CDP-diacylglycerol pyrophosphatase	*ushB*	1	3.98 %	61
Integration host factor alpha subunit (himA)	*ihfA*	1	12.12 %	57
Cel operon transcriptional regulator	*celD*	2	7.50 %	55
Chaperone protein DnaJ	*dnaJ*	2	6.86 %	53
30S ribosomal protein S14	*rpsN*	1	14.85 %	53
RNA polymerase sigma factor RpoS	*rpoS*	1	4.85 %	53
Putative ATP-binding protein SitB	*sitB*	1	8.42 %	53
Translation initiation factor IF2-alpha	*infB*	6	7.62 %	52
50S ribosomal protein L6	*rplF*	3	19.21 %	52


*A score of 50 was set as the cut off to report cross-linked proteins from *Salmonella*.*

### Receptor/Ligand Pairs Using Whole Cell Cross-Linking of Epithelial and Bacterial Cells

The ultimate utility of this approach is to find novel receptor/ligand partnerships during host/microbe association. The verification experiments with pure host proteins provided the basis to use whole *Salmonella* and host colonic cells *in vitro* to determine specific protein partnerships. After whole cell cross-linking was done, we observed SPTAN1 (host) to bind the protein produced from STM2699 (Fels-2 prophage tail) in *Salmonella*, while host proteins HSP90B1 and ACTN4 were bound to the protein from STM1956 (*fliA*) and STM4088 (*yiiU*) in *Salmonella*, respectively (**Table 4**). Of the four host proteins identified, SPTAN1 and HSP90B1 were previously reported to be involved in pathogenesis of *Salmonella* and *Listeria* (Cabanes et al., 2005; Ruetz et al., 2011; Martins et al., 2012), but no report of the microbial partner is known.

**Table 4:** Host/microbe binding partner proteins identified using whole cell cross-linking.

Estimate of Mw for gel spot (Da)	*S.* Typhimurium protein (LocusTag ID)	Unique peptides	PLGS score	Mw of bacterial protein (Da)	Caco-2 protein	Unique peptides	PLGS score	Host protein Mw (Da)
276,000	Putative Fels-2 phage tail-like protein (STM2699)	3	98.2	10,804	Spectrin, alpha, non-erythrocytic 1 (SPTAN1)	7	446.5	284,105
103,000	Flagellar biosynthesis factor FliA (STM1956)	3	87.4	27,456	Heat shock 90 kDa protein 1 beta (HSP90AB1)	13	651.7	83,212
94,000	Putative cytoplasmic protein (STM4088)	2	80.1	9,306	Tumor rejection antigen gp96 (HSP90B1)	9	394.6	92,411
					Actinin alpha 4 (ACTN4)	5	133	104,788


*PLGS score is calculated by the Protein Lynx Global Server (PLGS 2.2.5). A higher score indicates greater confidence in protein identity (Wright et al., 2009). Data presented represent the results obtained from three of five spots.*

### Impact of Putative Binding Partners on *in Vitro* Infection

**FIGURE 1:**
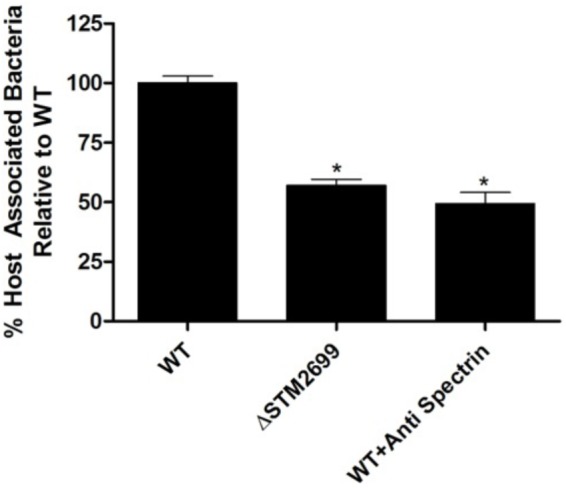
Host association with ΔSTM2699 mutant and anti-SPTAN1 antibody blocking. The amount of total host associated *Salmonella in vitro*. A significant reduction in adhesion (*p* < 0.05) between the wild type (WT) and treatment is indicated with “^∗^”. The error bars represent standard error of the mean from four biological replicates.

To examine the role of the putative whole cell binding receptor/ligand partners we conducted *in vitro* infection assays with *Salmonella* and colonic epithelial cells (Caco-2). We hypothesized that disrupting the receptor and the ligand partnerships would result in a reduction of *Salmonella* association *in vitro*. This hypothesis was tested using Ab-blocking of host receptors and genetic deletion of the bacterial ligand. Pre-treating Caco-2 cells with an α-SPTAN1 Ab significantly (*p* < 0.05) decreased the total host associated *Salmonella* by approximately twofold (**Figure 1**). Deletion of the Fels-2 prophage tail (STM2699) from the genome also resulted a significant twofold decrease (*p* < 0.05) in total *Salmonella* association. These observations confirm that SPTAN1 and STM2699 are cognate partners that mediate *Salmonella* association with colonic epithelial cells. STM2699 is annotated as Fels-2 prophage tail protein and has 100% identity to Fels-2 prophage tail proteins from other organisms (*E*-value = 5.8e-60) including nine other *S. ente*rica ssp. *enterica* genomes, *Enterobacter aerogenes* strain 682_EAER, *Pantoea* ssp. At9b, and *Erwinia billingiae*. This protein is controversial in its role in *Salmonella* pathogenicity (Andrews-Polymenis et al., 2004; Pickard et al., 2008), which is possible since it can be move between bacteria via horizontal gene transfer and transduction, but may play a role in host adaptation in *Salmonella* (Andrews-Polymenis et al., 2004). Shah et al. (2014) reported SPTAN1 to be involved in *Salmonella* virulence and used during host invasion during abiotic stress. With this positive identification of binding partners that have reported biological impact on *Salmonella* association, we proceeded to examine a more complex binding partner – APP – to determine if the identified binding partners play a role in *Salmonella* association.

**FIGURE 2:**
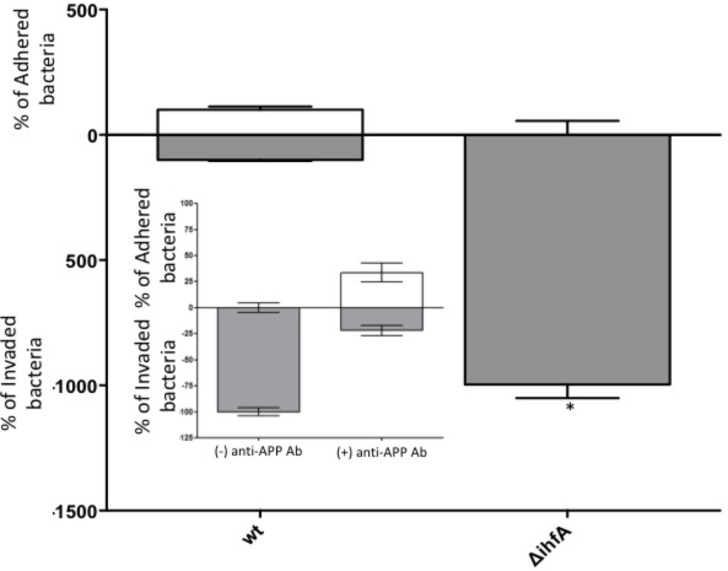
Host association with STM14_5216 mutant and anti-APP antibody blocking. The amount of adhered (white box) and invaded (gray box) *S.* Typhimurium and the integration host factor α (*ihfA*) deletion mutant with Caco-2 cells. The mean of tree biological replicates with error bars representing the SEM is shown. Significance is assigned as *p* < 0.05 and indicated with “^∗^”.

**FIGURE 3:**
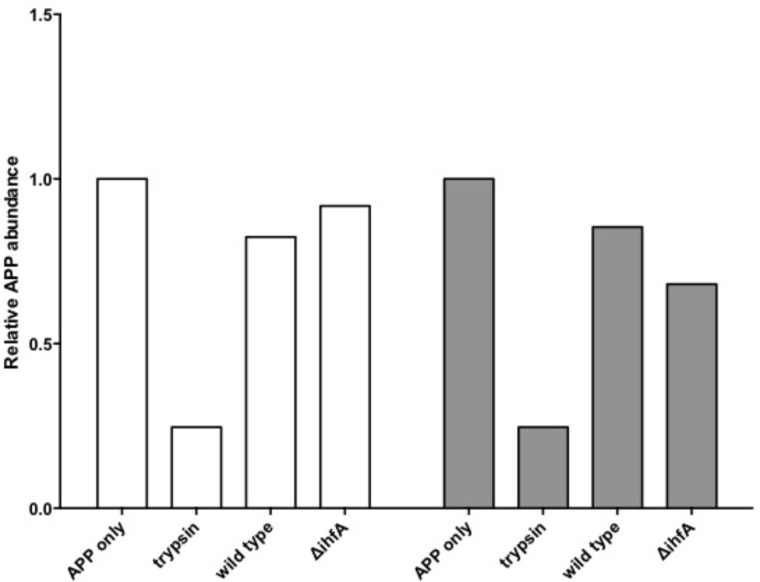
Amyloid precursor protein (APP) degradation by *S*. Typhimurium. Bars indicate the abundance of intact APP protein relative to the APP only control. Incubation of APP with trypsin functions as the positive control. White bars indicate APP degradation by the *Salmonella* cell pellet. Gray bars indicate APP degradation by the spent supernatant.

Unexpectedly, pre-treating the epithelial monolayer with anti-APP Ab prior to *Salmonella* association had no effect on host invasion (**Figure 2**), suggesting that this was not a primary binding partner for *Salmonella*. However, deletion of STM1339 (*ihfA*) resulted in a significant (*p* < 0.05) 10-fold increase in *Salmonella* host invasion. Since STM1339 (*ihfA*) regulates proteases in pathogenicity island 1 (SPI-1) in *Salmonella* (Mangan et al., 2006) we proceeded to examine possible digestion of APP by *Salmonella* during APP binding using the *ihfA*::Cm^R^ mutant. The ability of the mutant to digest APP did not differ from wild-type *Salmonella*; however, incubation of purified APP in the mutant spent supernatant resulted in a 25% decrease in intact APP (**Figure 3**). Deletion of STM1339 (*ihfA*) relieved the intricate regulation of the proteolytic cascade resulting in dysregulation of *Salmonella* protease system and export leading to a hypervirulent phenotype as observed previously in *Salmonella* (Heithoff et al., 2012). Taken together, these observations suggest that *Salmonella* produces a soluble protease(s) that digests APP during whole host cell association with *Salmonella* that results in a complex interaction that produces other biologically active end products.

## Discussion

Bacterial pathogens require intimate interaction with the host cell membrane in order to initiate host entry (Brandstaetter et al., 2012). To characterize host–microbe receptor/ligand partnerships at this resolution, a whole cell cross-linking method was developed using a cell impermeable cross-linking reagent, sulfo-SBED, to covalently link receptor/ligand cellular proteins that mediate host–bacteria association. By exploiting the molecular properties of Sulfo-SBED to cross-link membrane proteins between host and bacterial whole cells during adhesion, a new method was designed that identified cognate receptor/ligand partners. This strategy was anchored in biological associations to identify new molecular partnerships *a priori*. This is valuable in understanding how specific members of the microbiome interact with host cells to initiate host response beginning at the membrane that leads to molecular pathogenesis insights, possible drug targets, and vaccine candidates.

Validated of this approach was using Gram positive bacteria, *L. acidophilus* NCFM, and Gram negative bacteria, *Salmonella*, to demonstrate the utility among different types of bacteria. *L. acidophilus* NCFM binds specific extracellular matrix components (ECM), such as fibronectin during adhesion to host gastrointestinal cells but the bacterial fibronectin-binding proteins are not well characterized (Altermann et al., 2004). Using this cross-linking approach, previously identified as well as novel *L. acidophilus* fibrinogen ligands were identified. Ribosomal proteins are typically intracellularly localized but were observed bound to fibrinogen. This is previously observed due to their localization to focal adhesin complexes on the bacterial cell surface when induced with fibrinogen (**Table 1**; Chicurel et al., 1998). The novel *L. acidophilus* protein, CdpA, was also found to be bound to fibrinogen. Functional, informatic assessment of CdpA identified SLAP and FIVAR domains in CdpA (**Supplementary Figures S1**–**S4**) that are found in S-layer associated proteins were bound in the complex with fibronectin. Characterization of S-layer proteins reveals host association and host immunomodulatory roles of these proteins as well as occurrence of these domains in proteins that have host association roles (Johnson et al., 2013, 2015). The FIVAR domain was previously shown in the *Staphylococcus epidermidis* phage pSE109FN protein, Embp32, to bind fibronectin (Valentin-Weigand et al., 1993). Considering these findings and our observations of LBA0222 and CdpA (LBA0223) cross linked to fibronectin, and the ability of S-layer proteins to form a scaffold for host association in lactobacilli, it is likely that these two proteins work in conjunction to form a multi-protein complex to facilitate host association (Johnson et al., 2015). These results verify that the cross-linking approach is capable of identifying appropriate receptor/ligand molecules and is an effective method to discover partnerships in Gram positive bacteria. To examine the usefulness in Gram negative bacteria, *Salmonella* and *E. coli* interactions with pure host proteins were assayed before proceeding to a complex model.

While some ligands have been identified in *Salmonella* to be involved in host association, host receptors for *Salmonella* association have yet to be identified beyond a narrow range of molecules and effector proteins (Barnhart and Chapman, 2006; Keestra-Gounder et al., 2015). Our results showed that fibrinogen was cross-linked to *S.* Typhimurium Lpp, a murein lipoprotein expressed on the cell surface and previously reported as a virulence factor in *S*. sv Typhimurium. The protein mediates adhesion and invasion in a gut epithelial cell culture model (Sha et al., 2004). Persson et al. (2000) reported *csgA* fimbriae in *Salmonella* to be a fibrinogen binding protein, but this protein is not expressed in laboratory culture conditions (Humphries et al., 2003), explaining why it was not found cross-linked to fibrinogen in this study.

Fibrinogen binding is reported in *E. coli* (Shen et al., 1995), but no known fibrinogen binding proteins have been discovered. This study found UspB, a membrane protein in *E. coli* (Daley et al., 2005) that is required for ethanol tolerance, cross-linked to fibrinogen (**Table 2**). These results further validated the use of this technique with Gram negative and positive bacteria; therefore, we proceeded to find other partnerships that also mediated association during active infection conditions *in vitro*.

The APP is a transmembrane protein found on the basaolateral and apical surfaces of host cells, including colonic gastrointestinal epithelial cells (Puig et al., 2015). We observed appearance of the host binding protein for Aβ – beta amyloid binding protein (TM2D1) – during *Salmonella* infection *in vitro*, indicating an increase in Aβ production induced during infection (data not shown) and suggesting that APP is proteolytically digested by *Salmonella* during association with the host. Using the cross-linking method we identified 15 proteins from *Salmonella* that bound host APP (**Table 3**). Interestingly, each of those proteins contain protein domains involved in bacterial replication, stress response, DNA binding, or chromosome recombination.

Among the top hits identified as a *Salmonella* ligand for APP was integration host factor-α (IhfA) – a protein involved in the regulation of *Salmonella* SPI-1, a pathogenicity island that contains proteases used for infection (Queiroz et al., 2011). We focused on the regulation of proteases since we observed Aβ – beta amyloid binding protein (TM2D1) production in preliminary experiments. Both the whole bacterial cells and the spent supernatant of the Δ*ihfA* mutant hydrolyzed purified APP with the majority of proteolysis found in the spent supernatant (**Figure 3**). While *ihfA* itself is not reported to have proteolytic activity, it is part of a two-component transcriptional regulon that contains proteases involved in virulence (Queiroz et al., 2011). To further clarify this observation, the cross-linking result we examined the change in host association using the Δ*ihfA Salmonella* mutant (*ihfA*::Cm^R^). Dysregulation of proteolysis was observed in stationary phase cells and linked to altered host association. Disruption of pathogenicity islands, especially SPI-1, would lead to increased host association via using the *lon* protease in SPI-1 (Takaya et al., 2002; Bustamante et al., 2008). Deletion of Δ*ihfA* displayed a nearly 10-fold increase in host invasion (**Figure 2**), which is consistent with the observations of Takaya et al. (2002) who demonstrated the disruption of the SPI-1 proteolytic system led to a 10–40-fold increased invasion by *Salmonella* via an unexpected induction of *lon* (Takaya et al., 2002) as well as a more virulent phenotype in mice (Takaya et al., 2003). While this is in contrast to previous findings by Mangan et al. (2006), the use of early stationary phase cultures in our study, as opposed to exponential phase culture, likely contributed to the discrepancy between findings in wild type conditions, while deletion of Δ*ihfA* displayed the phenotype. Dysregulation of SPI-1 in the Δ*ihfA* mutant coupled with a SPI-2-induced stationary phase culture led to dysregulation of proteolytic capabilities and produced a hypervirulent organism that resulted in the observed 10-fold increase in host invasion (**Figure 2**).

After validation with pure host proteins the approach was modified to observe the host and microbial proteins cross-linked (**Table 4**), as evidenced by both proteins being identified from the same band in a SDS–PAGE gel. Of the four host proteins identified, two (i.e., SPTAN1 and HSP90B1) were previously reported to be involved in pathogenesis of *Salmonella* and *Listeria* (Cabanes et al., 2005; Ruetz et al., 2011; Martins et al., 2012). To confirm that this approach will find receptor/ligand pairs of functional significance, one of those partnerships (STM2699–SPTAN1) was examined to find that this association significantly altered *Salmonella* infection and is an example of a direct receptor/ligand partnership to control *Salmonella* association (**Figure 1**). Hence, the whole cell cross-linking method found pairs of receptors/ligand molecules with no prior knowledge of the specific mechanisms of association or invasion. In combination, cross linking to three different host receptors was confirmed chemically and biologically to establish this method is likely useful using whole cells with Gram-positive and Gram-negative bacteria.

## Conclusion

Microbial adhesion to host cells is mediated via protein–protein or a protein–glycan interactions (Kline et al., 2009; Arabyan et al., 2016; Park et al., 2016). The approach reported in this paper was validated by detecting previously reported receptor/ligand binding pairs. Subsequently, novel proteins that co-localized within 9–13 Å on the surface as well as other proteins that were in the same operon and likely to present at the same location on the bacterial surface. Expansion to cross-linking whole cells found known proteins with new host receptor partnerships, including APP, as well as complex interactions that led to digestion of APP during infection via disruption of the proteolytic cascade in *Salmonella*. This study developed a method to covalently bind interacting whole cells to identify proteins between live host cells and bacterial pathogens to rapidly discover new receptor/ligand pairs that mediate bacterial association and invasion. This approach is applicable to any host/microbe interaction that is mediated by protein/protein interactions and has the potential to uncover direct partnerships and complex protein digestion processes. To the best of our knowledge this is the first report of whole cells cross-linking to discover host–microbe protein–protein interactions during the native infection process.

## Author Contributions

PD and BW planned the experiments. PC, PD, and DC conducted cross-linking and proteomic analysis and mass spectrometry. BW, PC, and PD wrote the manuscript. PC and JS constructed the gene deletion mutants.

## Conflict of Interest Statement

The authors declare that the research was conducted in the absence of any commercial or financial relationships that could be construed as a potential conflict of interest.

## Supplementary Material

FIGURE S1 Example of two 2D gels for non-reduced **(A)** and reduced **(B)** gels used to obtain protein spots for ID. Circled locations show areas that were a combination of bacterial and host proteins. Numbers indicate specific spots that were picked for ID. Comparing those locations between gel **(A)** and gel **(B)** indicated that those were cross-linked and released in reducing conditions.

FIGURE S2 Phylogenetic tree of amino acid sequences alignments of CdpA and its homologs. Phylogenetic tree of amino acid sequence alignments of CdpA, Lba0222, and homologs. Asterisks indicate the proteins identified in cross-linking assay.

FIGURE S3 Unique *cdpA* gene neighborhood structure and CdpA protein network analysis. **(A)** Functional protein network prediction of CdpA and LBA0222. Each unique protein network partner is indicated by a colored sphere. Blue lines joining each sphere indicate predicted partnership by text mining. Green lines joining each sphere indicate predicted partnership by gene neighborhood. **(B)** *cdpA* gene neighborhood is indicated by the colored tabs next to *Lactobacillus acidophilus*. Gene neighborhood uniqueness is indicated by the absence of similar tabs next to surrounding species and genera.

FIGURE S4 LBA0222 and CdpA (LBA0223) protein domain analysis. Dark and light purple arrows indicate operon membership. Domain analyses are listed in the tables below each gene.

TABLE S1 PCR primer sequences used in this study.
