# Supplementary figures and images for: Whole Cell Cross-Linking to Discover Host–Microbe Protein Cognate Receptor/Ligand Pairs
Source: Front Microbiol. 2018 Jul 19;9:1585. doi: 10.3389/fmicb.2018.01585 (PMC6060266; doi:10.3389/fmicb.2018.01585)

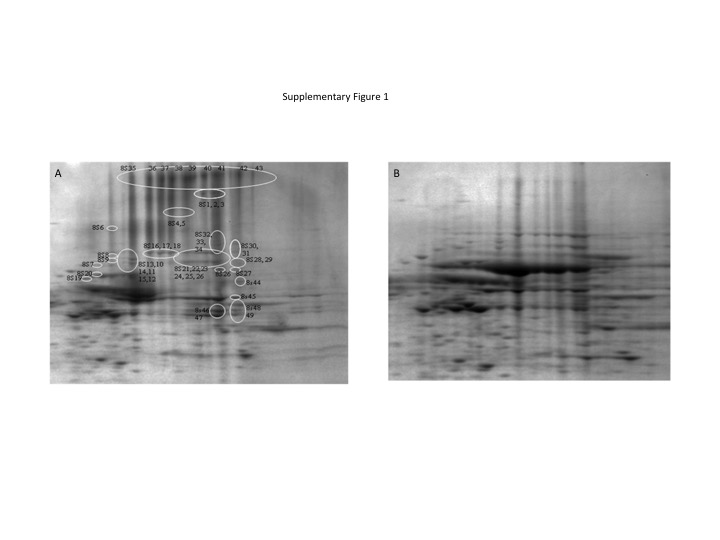

Supplement: FIGURE S1 — Example of two 2D gels for non-reduced (A) and reduced (B) gels used to obtain protein spots for ID. Circled locations show areas that were a combination of bacterial and host proteins. Numbers indicate specific spots that were picked for ID. Comparing those locations between gel (A) and gel (B) indicated that those were cross-linked and released in reducing conditions. [file Image_1.JPEG]

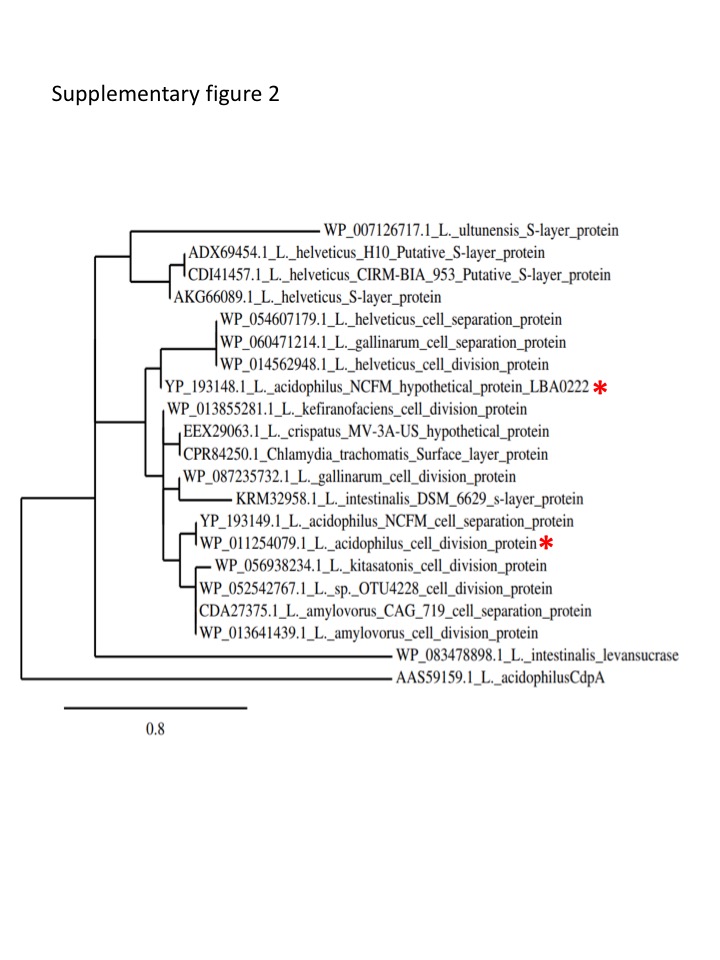

Supplement: FIGURE S2 — Phylogenetic tree of amino acid sequences alignments of CdpA and its homologs. Phylogenetic tree of amino acid sequence alignments of CdpA, Lba0222, and homologs. Asterisks indicate the proteins identified in cross-linking assay. [file Image_2.JPEG]

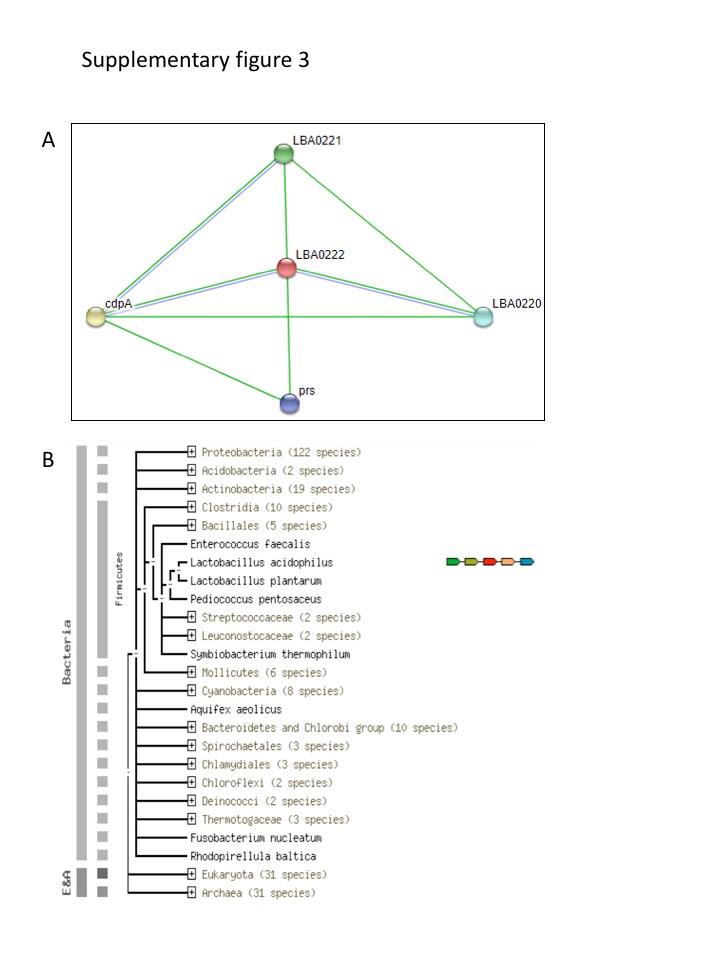

Supplement: FIGURE S3 — Unique cdpA gene neighborhood structure and CdpA protein network analysis. (A) Functional protein network prediction of CdpA and LBA0222. Each unique protein network partner is indicated by a colored sphere. Blue lines joining each sphere indicate predicted partnership by text mining. Green lines joining each sphere indicate predicted partnership by gene neighborhood. (B) cdpA gene neighborhood is indicated by the colored tabs next to Lactobacillus acidophilus. Gene neighborhood uniqueness is indicated by the absence of similar tabs next to surrounding species and genera. [file Image_3.JPEG]

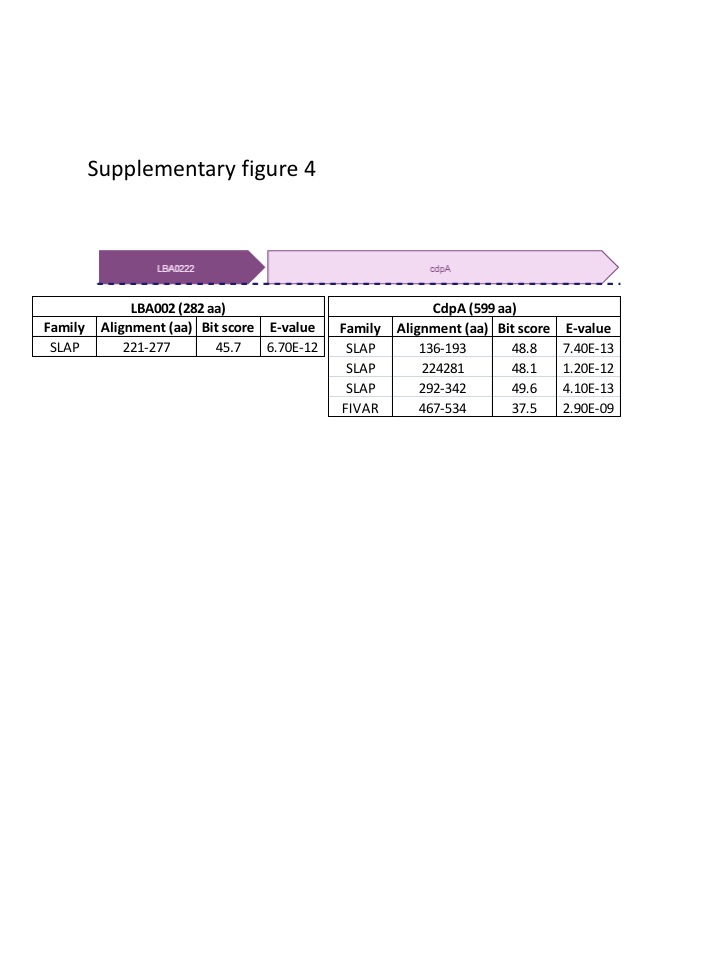

Supplement: FIGURE S4 — LBA0222 and CdpA (LBA0223) protein domain analysis. Dark and light purple arrows indicate operon membership. Domain analyses are listed in the tables below each gene. [file Image_4.JPEG]
