# Supplementary material for: Whole Cell Cross-Linking to Discover Host–Microbe Protein Cognate Receptor/Ligand Pairs
Source: Front Microbiol. 2018 Jul 19;9:1585. doi: 10.3389/fmicb.2018.01585 (PMC6060266; doi:10.3389/fmicb.2018.01585)
Supplement: TABLE S1 — PCR primer sequences used in this study. [file Table_1.docx]

Supplementary Table 1. PCR primer sequences used in this study.

| Primer Name | Sequence (5'-3') |
| --- | --- |
| STM2699 P1 | CCTTAACGGCGCGGGCAGCCGCGCCAGTAT TTCATTAACAGGATACGAACGTGTAGGCTGGAGCTGCTTC |
| STM2699 P2 | TGGCGACCAGTGAAAGATGG TGGCGATATCCGCCACAAGATCATCAATCGATGGGAATTAGCCATGGTCC |
| STM2699 F | ATGAGCGACAAGCTGACTGA |
| STM2699 R | GCCCACGTCCATATCCATAA |
| STM2699 J1 | CAGGATACGAACGTGTAGGC |
| STM2699 J2 | AAGATCATCAATCGATGGGAAT |
| STM14_1626 P1 | TGTAGAGGCATTAAAAGAGCGATTCCAGGCATCATTGAGGGATTGAACCTGTGTGGCTGGAGCTGCTTC |
| STM14_1626 P2 | GCAGCGTATCTGCCGCAATACACCCTGATGGATGTTATGCCTGGATCTGAATGGGGAATTAGCCATGGTCC |
| STM14_1626 J1 | GAGGGATTGAACCTGTGTAGGCTGG |
| STM14_1626 J2 | GCCTGGATCTGAATGGATGGAATTAGCC |
| STM14_1626 F | GGGATGCCAAAGAACTGGTTGA |
| STM14_1626 R | GCTCTTTAACTTCTGCCCGGG |
